# Comment on Liu et al. Hemodialysis Treatment for Patients with Lithium Poisoning. *Int. J. Environ. Res. Public Health* 2022, *19*, 10044

**DOI:** 10.3390/ijerph20105843

**Published:** 2023-05-17

**Authors:** Michael Ott, Ursula Werneke

**Affiliations:** 1 Department of Public Health and Clinical Medicine–Medicine, Umeå University, 901 87 Umeå, Sweden; 2 Sunderby Research Unit, Department of Clinical Sciences, Division of Psychiatry, Umeå University, 901 87 Umeå, Sweden

In a recent article, Liu and colleagues presented a case-series of patients with lithium poisoning, with special emphasis on hemodialysis [1]. In their table 1, they cited our previous study [2], reporting a mortality rate of 4.3%. However, in our study, as already explicitly stated in the published paper, there were no fatalities related to lithium intoxication. As the authors correctly discuss, the best treatment of the different scenarios of lithium intoxication remains subject to debate. To exert its harmful and potentially life-threatening effects, lithium must enter the cell. In early acute intoxications, serum lithium levels (s-Li) reflect extracellular concentration. In chronic intoxications, however, s-Li most likely reflects intracellular concentration. Chronic intoxications often result from reduced renal excretion when lithium dosage has not been adapted [2]. Lithium is completely filtered by the glomeruli and not to 80%, as suggested by Liu et al. Then, 80% of the filtered lithium is reabsorbed in the tubuli [3]. Lithium reabsorption roughly follows sodium reabsorption. Saline infusions both prevent hypovolemia and minimize lithium reabsorption. The use of hemodialysis in the setting of chronic intoxication is mainly a question of the severity of symptoms and the residual renal function. 

In acute or acute-on-therapeutic intoxications, the clinical decision to dialyze is far more complex. While blood concentrations of lithium are high, symptoms often have not yet developed. Once lithium has entered the intra-cellular compartment, the window of opportunity for the removal of lithium has passed. We compared a French study by Vodovar et al. [4] with our own Swedish data, using the same cut-offs [5]. Patients in Sweden experienced significantly lower rates of coma and cardiovascular failure. The proportion of patients dialyzed was significantly higher, especially in the group with lithium levels ≤5.2 (4% vs. 31%). Figure 1 illustrates the pathophysiological background of the two scenarios (adapted after [5]). To decide whether to dialyze in the setting of acute/acute-on-chronic intoxication, the treating physician needs to consider how much lithium is on its way to eventually enter the cells. Hemodialysis is a safe procedure. When available, we advocate its early use because, once lithium has entered the cells, tissue damage may be irreversible. 

## Figures and Tables

**Figure 1:**
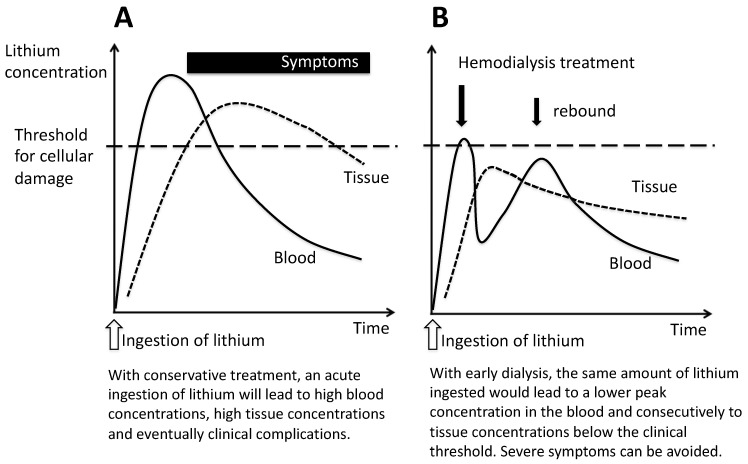
Scenarios of severe acute or acute-on-therapeutic intoxication with (**A**) and without hemodialysis (**B**).
